# Otorhinolaryngological Problems in Mucopolysaccharidoses: A Review of Common Symptoms in a Rare Disease

**DOI:** 10.3390/brainsci14111085

**Published:** 2024-10-29

**Authors:** Anna Waśniewska-Włodarczyk, Renata Pepaś, Oskar Rosiak, Wiesław Konopka

**Affiliations:** 1 Department of Otolaryngology, Polish Mother’s Memorial Hospital Research Institute, 93-338 Lodz, Poland; renatapepas@interia.eu (R.P.); orosiak@gmail.com (O.R.); wieslaw.konopka@umed.lodz.pl (W.K.); 2 Department of Paediatric Didactics, Medical University of Lodz, 90-419 Lodz, Poland

**Keywords:** mucopolysaccharidoses, otorhinolaryngology, otology, otolaryngology, ear, nose, throat

## Abstract

Background: The mucopolysaccharidoses (MPSs) are very rare lysosomal diseases. MPSs belong to inherited diseases; however, newborns are usually asymptomatic. A deficiency of one of the enzymes, which is responsible for glycosaminoglycan (GAG) catabolism, results in the accumulation of this material. GAGs lead to progressive damage to tissues. More than 90% of patients with MPS suffer from otitis media with effusion or recurrent otitis media, craniofacial dysmorphia, obstructive sleep apnea, different types of hearing loss, and progressive upper and lower airway dysfunction. Patients visit otolaryngologists often before the diagnosis of MPS. Thus, the awareness of symptoms of MPS is crucial for otolaryngologists and pediatricians. The earlier the diagnosis is made, the more effective treatment is. Ineffective or delayed treatment leads to premature death. Two principal treatments for MPS are currently available: hematopoietic stem cell transplantation (HSCT) and enzyme replacement therapy (ERT). In recent years, there has been a growing interest in gene therapy as a potential treatment for patients with MPS. Mortality in patients with MPS typically occurs during childhood and early adolescence as a consequence of upper and lower respiratory diseases. Methods: This systematic review is based on papers available in the following scientific databases: MEDLINE (via PubMed), Web of Science, Scopus, and the Cochrane Library. Results: After screening, 72 articles met our inclusion criteria. Conclusions: It is of paramount importance that otolaryngologists are involved in this field. This narrative review examines and synthesizes the otolaryngologic issues encountered in patients with MPS.

## 1. Introduction

The mucopolysaccharidoses (MPSs) belong to the rare lysosomal storage diseases, and global incidence varies between 1.56 and 4.8 per 100,000 live births depending on the region [1,2,3]. The first case of male twins with MPSs was first described in 1917 by Major Charles Hunter [4]. Since then, multiple MPS types and subtypes have been categorized (Table 1). MPSs are inherited deficiencies of enzymes, which are essential for the degradation of the group of extracellular heteropolysaccharides, glycosaminoglycans (GAGs). GAGs are involved in inflammation processes, neurodevelopment, tumor progression, and, what is most important, they constitute the structural support for tissues [5,6]. Progressive accumulation of GAGs in lysosomes causes multiple disturbances of the skeleton, eyes, ears, cardiopulmonary system, and neurocognitive functions. The precise symptoms depend on the specific defective enzyme. Also, types and subtypes differ in the intensity of their symptoms. MPS I Hurler syndrome and MPS IIA are severe diseases that develop during the first year of life, and when untreated usually result in death in the first decade of life. Whereas patients with MPS I Sheie syndrome, characterized by a longer lifespan, typically die at the age between 25 and 30 years old. Patients with MPS VI slowly progress, but most patients do not reach the third decade of life. MPS VII is often connected with hydrops fetalis; fetuses die shortly after birth or are stillborn. Few patients with MPS VII survive into adulthood [7]. Newborns with MPS are usually asymptomatic. The first signs of the disease may be noticed in infants or small children. However, the diagnosis is usually delayed, which also affects the initial time of the treatment (hematopoietic stem cell transplantation (HSCT) and enzyme replacement therapy (ERT)). The imperfection of these treatment methods led to studies on gene therapies, but they are still in the clinical trials phase. Severe types with early onset may be diagnosed at a younger age; unfortunately, it might be too late to effective treatment with HSCT and ERT. The odds for successive treatment for patients with MPS could be higher due to neonatal screening; unfortunately, only a few countries have introduced it. Patients make multiple visits to different specialists for their symptoms. Isolated symptoms, which are common among the general population, often do not raise concern. Therefore, making an early diagnosis of MPS is rare. Diagnosis usually occurs when one of the specialists takes a comprehensive history and discovers the complexity of the patient’s problem [5,6]. The most common cause of death depends on the type of MPS. In MPS I, II, IV, and IV, respiratory and/or cardiovascular problems are dominant; in MPS III, neurological deficits are dominant; and in MPS VII, hydrops fetalis is dominant [8]. Over 90% of patients with MPS suffer from ear, nose, and throat (ENT) problems, thus, ENT doctors are often the first specialists for these patients [2,9,10,11,12,13]. The knowledge about MPS among ENT doctors and pediatricians may be highly significant since the effectiveness of treatment depends on the stage of the disease [7,14].

## 2. Materials and Methods

This review is based on papers available in the following scientific databases: MEDLINE (via PubMed), Web of Science, Scopus, and the Cochrane Library. The search strategy included the following keywords: “otorhinolaryngology”, “otolaryngology”, “ENT”, “laryngology”, “mucopolysaccharidosis”, and “MPS”. Databases were searched before 30 June 2024. We used the following inclusion criteria: (1) originals, review papers, and case reports; and (2) subjects of papers on MPS and otorhinolaryngologic problems. The exclusion criteria included the following: (1) conference reports and letters to the editors; and (2) papers in languages other than Polish or English. There was no time window for the studies. Two authors independently reviewed the titles and abstracts.

## 3. Results

After screening, 72 articles met our inclusion criteria. All articles are described in the Supplementary Material. The comparison of the original articles and ENT symptoms is summarized in Table 2.

### 3.1. Craniofacial Dysmorphia

**Figure 1:**
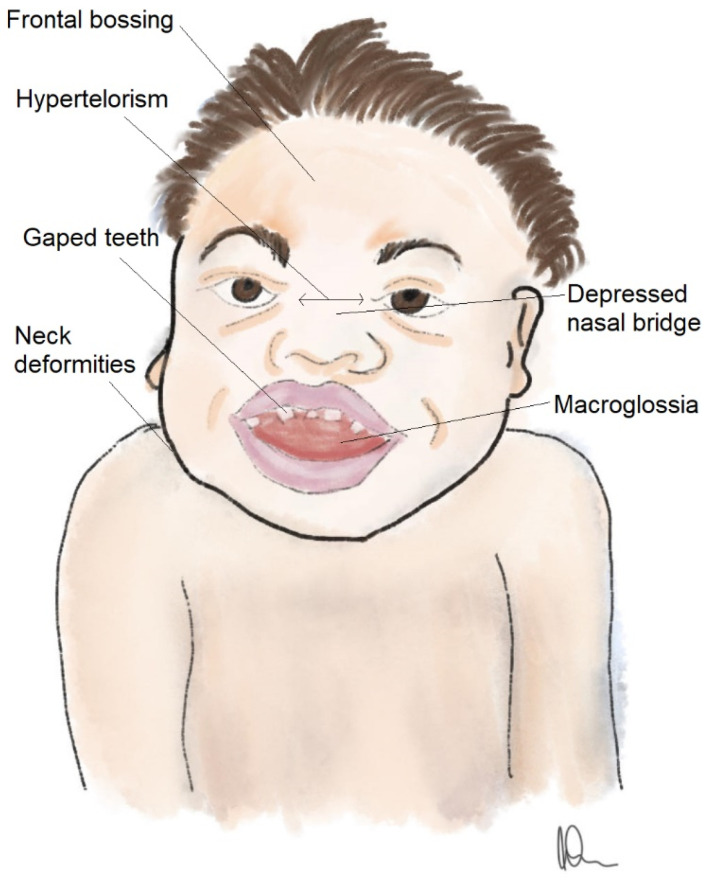
Craniofacial dysmorphia in patients with MPS. Author: A.W.-W.

The degree of skull and facial dysmorphia varies not only between types of MPS but also between subtypes. Usually, patients are characterized by bossing of the frontal bone, retrusion of the midface, a deep cranial fossa that narrows the nasopharynx, and a flattened nasal bridge [Figure 1]. Patients with MPS suffer from small nasal passages due to thick mucus membranes, irregular development of facial bones, and increased secretions. Oral abnormalities include thickening of the lips, macroglossia, high-arched palate, and malocclusion traits, caused by dental irregularities and defects of the mandibular condyle. The eruption of some teeth is delayed. Patients with MPS are characterized by supernumerary teeth, taurodontism, root dilaceration, and impacted teeth [45,46,47]. Teeth are usually small, and spaces between them are broad due to the gingival overgrowth. According to panoramic radiograph studies among patients with MPS, one of the most common maxillomandibular alterations are dentigerous cysts [48,49]. This kind of cyst is usually asymptomatic but may lead to resorption of the tooth and bone [48]. The alveolar ridges may be flattered or enlarged. Patients may also suffer from different enamel defects. Dental and oral abnormalities have not only aesthetic consequences but also affect chewing and quality of life [45]. Macrocrania is usually noticed before the first birthday in patients with MPS types I, II, III, IV, VI, and VII. In MPS I, facial dysmorphia may be visible from six months old, in types II and VI between 9 and 18 months, whereas in MPS III it is visible starting at two years old [50,51].

### 3.2. Upper Airway Obstruction

**Figure 2:**
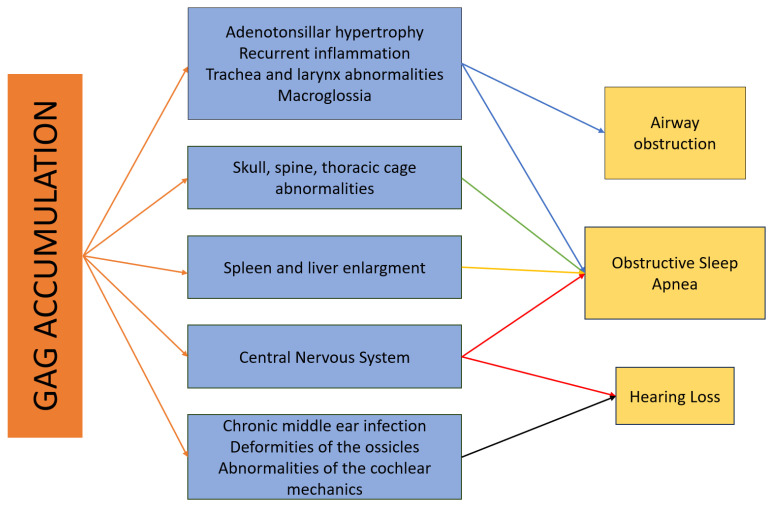
Otolaryngological problems due to accumulation of GAG.

Patients with MPS types I, II, IVA, VI, and VII are particularly at risk of respiratory failure [25]. Apart from the high accumulation of GAG in tissues, more than 50% of patients suffer from recurrent ear and throat infections [52]. Among upper airway problems, we can list adenoid and tonsillar hypertrophy, macroglossia, nasal, pharyngeal, and laryngeal abnormalities, increased mucus secretions, recurrent respiratory infections, obstructive sleep apnea, and tracheomalacia [Figure 2].

#### 3.2.1. Adenotonsillar Hypertrophy

Regardless of the type of MPS, most of the patients suffer from adenoid, tonsillar hypertrophy, or both. According to the literature, prevalence varies between 35% and 96% [12,13,18,26,53,54,55]. GAGs are also responsible for the recurrence of adenoid hypertrophy, which is more common among patients with MPS (56% of cases) than the general population (0.55–1.5% of cases) [53]. Apart from the GAG accumulation in lymphatic tissues in patients with MPS, recurrent infections are also responsible for tonsillar pathology [9]. Adenoid and tonsil surgeries are one of the most common surgical procedures in patients with MPS [26,27]. Frequently, diagnosis of MPS is made after tonsillectomy or adenoidectomy [28]. This observation encouraged Keilmann et al. to study the removed tissue after adenoidectomy and/or tonsillectomy of 177 patients suffering from MPS to find possible markers of disease for early diagnosis. The authors claim a specificity of 92% and a 100% sensitivity in diagnosing MPS [53]. Adenoidal and tonsillar surgeries in patients with MPS are more challenging than usual. Neck instability is common among patients with MPS; thus, the surgeon should not extend the neck. Temporomandibular joint and neck stiffness in different MPS types narrows the operating field [9,26,47]. According to Nayak et al., endoscopic adenoidectomy in patients with MPS may be a simpler solution to these problems [56]. Unfortunately, patients with MPS experience more complications after surgery in comparison with the general population. Patients are at risk of airway oedema, postoperative hemorrhage, a longer length of stay, more expensive treatment, and failure to extubate [54,57,58]. Pal et al. analyzed adenotonsillar samples from patients with MPS types I, IVA, and VI who were treated with ERT. They reported GAG accumulation in adenoids and tonsils, despite treatment with ERT [59].

#### 3.2.2. Larynx and Trachea Abnormalities

In patients with MPS, airway problems develop through adenoids, tonsils, larynx, and trachea, up to the bronchi. In the larynx, supraglottic tissues are often enlarged, and vocal folds and ventricular bands are thickened. Deformities of the epiglottis and cricoid cartilages are also noticed [29,60,61]. Accumulation of GAG in the aryepiglottic folds and arytenoid cartilages may result in the prolapsing of these structures and lead to airway obstruction [9,54]. The tracheal lumen can be narrowed, tortuous, and occluded due to the overgrowth of soft tissue and enlargement of cartilage cells. In adolescents, accumulation of mucopolysaccharides may create “tumors”, leading to stenosis of the airways [11,30,47,62]. Muhlebach et al. reported a study describing 105 bronchoscopies, which were performed in 31 patients with MPS types I, II, III, IV, and VI. In 46% of cases, they diagnosed bronchomalacia, and in 31%, laryngomalacia. As they noticed, airway dysfunction can occur in early childhood [63]. Theroux et al., in their cohort study, which analyzed 92 patients with MPS IVA, noticed that tracheal narrowing worsens in older patients; however, the progression seems to decelerate after 20 years of age [64]. Another factor that has an impact on the trachea is skeletal deformities, especially spinal. The disproportion in growth between the neck, chest, and trachea leads to tracheal kinking [25,65]. Compression of the spinal cord and instability of the spine affect the phrenic nerve, causing dysfunction of the respiratory system [25]. The obstruction of airways in patients with MPS can manifest as cough, dyspnea, stridor, cyanosis, and dysphagia [29]. Lesions located inside the trachea may be treated with the use of Nd-Yak or CO_2_ laser via a rigid bronchoscope [66,67]. Sometimes the only solution for patients is tracheostomy; however, this procedure should be preceded by chest computed tomography to detect and avoid the stenosis distal to the carina [47,67,68,69]. According to the literature, up to 16% of children with MPS need a tracheostomy [11,47,60]. Bronchial secretions may also be successfully managed by tracheostomy. Nonetheless, changes in the auto-positive end-expiratory pressure (auto-PEEP) after tracheostomy may reveal the collapsing of the airway, which is a result of tracheomalacia and bronchomalacia. Tracheotomy in patients with MPS carries an increased risk of intrastomal tracheal stenosis, stomal narrowing, wound infection, and granulation formation [47,68,69,70,71]. Difficulties in the lower airway or the coexistence of stenosis and malacia require different treatments. Thus, many studies [19,67,72] reported the use of tracheal stenting in patients with MPS [73]. This solution assures permanent open airways [67]. The new and interesting idea of treatment for tortuous trachea was reported by Frauenfelder et al. They successfully performed tracheal resection and limited manubriectomy in patients with MPS type IVA [65].

#### 3.2.3. Obstructive Sleep-Disordered Breathing (OSDB)

Although obstructive sleep-disordered breathing (OSDB) is a symptom of various problems in patients with MPS, it requires special consideration of ENT doctors and pediatricians. OSDB may occur as a simple snoring to obstructive sleep apnea syndrome (OSA) [31]. The pathogenetic mechanism of OSA is based on the anatomical constriction of upper airways and dynamic alterations occurring during sleep, which involve increased airflow resistance, muscle relaxation, and a heightened likelihood of obstruction in an already narrowed air passage. Usually, patients with OSA are characterized by snoring, mouth breathing, non-restorative sleep, and apnea [32]. OSA may affect up to 89% of patients with MPS [26,32,33]. OSA affects patients with MPS III the least. Apnea index is significantly higher in children than in adults (*p* = 0.03) [34,74]. In addition to adenotonsillar hypertrophy, larynx, and trachea abnormalities, OSA may be caused by spleen and liver enlargement, which limits diaphragm excursion. Furthermore, the shape of the chest wall is deformed due to kyphoscoliosis and pectus carinatum, which affects pulmonary function [75]. Cervical spine instability and odontoid dysplasia may compress the spinal cord and, as a consequence, may induce central sleep apnea [76]. Patients from the general population with OSA may develop learning difficulties, behavioral problems, and cardio-respiratory failure. Patients with MPS may experience these problems independently of OSA, but the presence of OSA may exacerbate them [33]. All patients with a diagnosis of MPS need a polysomnography examination, even before obstructive symptoms [32,35,71]. As the first line of treatment, ENT doctors suggest the removal of the obstruction by surgery [63,69]. If local surgical airway procedures are insufficient, continuous positive airway pressure (CPAP) may be implemented. Unfortunately, CPAP is less beneficial for patients with behavioral disturbances or in progression of airway disease. In patients with MPS with hypoxemia despite CPAP use, non-invasive ventilation (e.g., bi-level positive airway pressure) may be considered. Further steps involve tracheotomy and stents [36,54].

### 3.3. Vestibulocochlear Abnormalities

#### 3.3.1. Hearing Loss

Disturbances of auditory function occur in close to 100% of patients with MPS [37,38,39,77]. So far, there is no information regarding hearing loss in MPS IVB and MPS IX [77]. According to Lenka et al., the median age of diagnosis of hearing impairment in patients with MPS is 4.5 years, which is a crucial age in speech development [2]. Patients may suffer from conductive hearing loss (CHL), sensorineural (SHL), or mixed. The conductive type is an effect of bone chain deformities, seromucous otitis, or difficulties in ossicular conduction, caused by conditions similar to arthropathy or otosclerosis. The etiology of sensorineural hearing loss is still uncertain. However, there are a few working hypotheses. One of them claims that SHL in MPS results from the accumulation of GAG in neural structures such as the brain stem, vestibulocochlear nerve, and cochlea. Owing to the progress of hearing loss in patients with MPS and the change from CHL to SHL with age, there might be other factors of SHL [13,24,40,54,77]. This statement was supported by Kariya et al. They examined 11 temporal bones from 6 patients with MPS IH and compared them with 14 temporal bones from 7 control cases. The amount of cochlear hair cells in patients with MPS IH was significantly decreased in comparison with healthy controls [78]. Absence of response in DPOAEs suggests dysfunction of outer hair cells, whereas changes in wave I of ABR indicate a smaller number of inner hair cells [79]. The deficit cognitive function and challenges in general anesthesia limit performing a crucial auditory examination, such as auditory brainstem response (ABR) [2,42]. Changes caused by the accumulation of GAG are visible in temporal bone computed tomography. Cho et al. examined 32 ears of patients with MPS II. They reported thickening of the external auditory canal in 50 % of cases and the tympanic membrane in 65% of cases [43]. In addition, abnormalities of the mastoid process were visible. Regardless of the reason for computed tomography of the head in patients with MPS, an accurate evaluation of ear structures should be conducted. Otitis media with effusion (OME) in patients with MPS is more common than in the general population as a result of adenoid hypertrophy, GAG accumulation in middle ear fluid, and cranial deformities, which affect Eustachian tubes [2]. CHL as a secondary symptom demands the removal of the primary cause. First-line treatment is usually adenoidectomy with the insertion of middle ear ventilation tubes. If short-term ventilation tubes are insufficient or the procedure needs multiple insertions, ENT doctors should consider permanent t-tubes [80]. However, not every MPS patient with OME can be operated on due to their airway obstruction problems and inability to be intubated. In these cases, receiving hearing aids, as a safer but unconventional approach, may be recommended [44]. The main treatment for patients with MPS with SHL is hearing aids, like cochlear implants. ENT surgeons should be aware of possible atypical findings in the anatomy of patients with MPS, such as vessel overgrowth. What is more, the multidisciplinary team should consider if the risk of anesthesia and surgery is not greater than the benefits of the cochlear implant in this group of patients [41]. Several reports have described improvement in hearing after hematopoietic stem cell transplantation (HSCT), especially when therapy was implemented before 25 months of age [77,81]. According to van der Broek et al. [82], in many cases, CHL may still occur soon after HSCT, and SHL occurs at longer follow-up. The mortality rate of HSCT is relatively high; it may even be 11% [81]. Contrary to HSCT, ERT in patients with MPS is a safe and effective treatment [83]. According to Hong et al. and their animal model, if ERT is introduced before hearing loss, it is possible to prevent CHL, whereas SHL may not be prevented [84]. Exact results of the ERT on hearing are still dubious [77,85,86]. Further studies investigating the long-term influence of ERT and HSCT on hearing loss in patients with MPS are needed. In MPS, proper speech therapy is crucial, and patients may benefit regardless of the cognitive impairment [43].

#### 3.3.2. Vestibular Abnormalities

Ohlemiller et al. performed a histopathologic examination of the MPS VII in mice [87]. They confirmed lysosomal storage in the cristae and vestibular maculae, as well as in the hair and supporting cells. Yigit et al. performed computerized dynamic posturography on 10 patients with MPS and 9 healthy people [88]. A statistically significant difference was noticed between the two groups in the sensory analysis test and the adaptation test.

## 4. Discussion

Multiple ENT problems may occur in patients with MPS. First, every otolaryngologist and pediatrician should carefully look at the patient. Hirsutism, hypertelorism, bossing of the frontal bone, retrusion of the midface, and flattened nasal bridge are not usually observed in children but are characteristic for patients with MPS. Otolaryngologists should be especially aware of this disease due to life-threatening respiratory disorders, which develop in all types of MPS and are usually progressive. Airway dysfunction results in early mortality, often in childhood [11]. Deposits of mucopolysaccharides may be not only located in the tonsil and adenoids but also in the tonsillar fossa. This is why the coblation intracapsular tonsillectomy is not recommended in this group of patients. Keilmann et al. [53] noticed that examination of adenoid or tonsil tissue may be a possible option for the diagnosis of MPS. However, the histopathology of all removed tissues after this surgery is not a cost-effective solution. Accumulation of GAG in adenoids and tonsils [31] suggests that patients should still be under the control of ENT doctors, even after ERT. According to all the above airway problems, patients with MPS are named “the worst airway problem in pediatric anesthesia” [89]. That is why the endoscopic evaluation of the larynx, trachea, and bronchi is recommended to diagnose the problem, as well as before general anesthesia to omit difficulties during surgery [90]. Furthermore, the presence of an otolaryngologist should be considered at every anesthetic induction to perform a tracheotomy if the orotracheal intubation is not achievable. Surgery that is routine for the general population, such as adenectomy or hernioplasty, in patients with MPS may be life-threatening due to their airway problems [47,91]. Diagnosis and examination of hearing impairment in patients with MPS is difficult. It should be noted that speech audiometry may present worse results than pure tone audiometry. The speech audiometry demands good understanding and verbal development, but patients with MPS often characterize with retardation and delay of verbal abilities. The selection of the appropriate hearing examination is crucial in the diagnosis of hearing loss.

Only a few studies have investigated vestibular abnormalities in patients with MPS. The lack of information in this area is probably caused by progressive cognitive impairment and physical disability, which makes it difficult to perform a proper postural balance function examination. Findings reported by Ohlemiller et al. [87] support recommendations that apart from routine hearing assessments, patients with MPS should also receive balance and vestibular system examinations. Most patients with MPS require surgical treatment. Nevertheless, due to multiple difficulties in the public health system, not all patients who need this are operated on.

## 5. Limitations

Despite a deep analysis of ENT problems, this study has limitations. MPSs are rare diseases and are sparsely described in the literature. The lack of sufficient studies significantly hinders the formulation of specific hypotheses and guidelines. Moreover, this review does not meet the criteria of a systematic review. Thus, probably we did not mention all of the studies regarding MPSs and otolaryngologic problems. Even though we prepared tables with comparisons of multiple original articles, the narrative review lacks a stringent analysis or results. 

## 6. Conclusions

This review highlights the complexity of otolaryngologic symptoms in all types of MPS. ENT doctors and pediatricians are important specialists providing care to patients with MPS. This review, in the hands of ENT specialists or pediatricians, may lead to early diagnosis and treatment of patients with MPS, saving their health and life. Due to the high incidence of hearing loss in patients with MPS, this symptom should be alarming, especially in small children. Airway obstruction should be suspected in all types of MPS; some of them may be severe and potentially life-threatening. Furthermore, sleep disorders associated with respiratory problems should be suspected and evaluated early. More studies are needed to verify the impact of ERT and HSCT on different ENT symptoms in various MPS types.

## Figures and Tables

**Table 1:** Types and subtypes of MPS.

MPS Type	Gene	Common Name	GAG	Symptoms Other Than ENT
MPS I	*IDUA*	Hurler, H–S, Sheie	DS, HS	MPS I Hurler (severe): - Rapid progress; - Developmental delay; - Skeletal disorders; - Craniofacial dysmorphia; - Hepatic and cardiac disease; - Death usually within the first year of life [15]. MPS I H-S Intermediate): - Diagnosis between 2 to 6 years; - Hepatosplenomegaly; - Skeletal abnormalities similar to MPS IH; - Thickening of the meninges; - Mild cognitive impairment; - Death is usually in early twenties [15]. MPS I Sheie (mild): - Also called MPS type V [16]; - Symptoms similar to other MPS I subtypes; - Patients keep normal intelligence; - Death between 25–30 years old [15].
MPS II	*IDS*	Hunter	DS, HS	- Develop between 18 months and 4 years; - Inguinal and umbilical hernia; - Bone dysplasia, joint stiffness; - Behavioral problems; - Severe cognitive impairment; - Neurodegeneration; - Cardiac disease; - Death at the end of the second decade of life; - Might be divided in two types IIA (severe) and IIB (moderate) [17].
MPS III	*GNS*, *HGSNAT*, *NAGLU*, *SGSH*	Sanfilippo A,B,C,D,E	HS	- Severity of symptoms varies between subtypes of MPS III, the most severe are in MPS IIIA; - Aggressiveness; - Intellectual deterioration; - Dementia; - Facial dysmorphia; - Seizures; - Cardiac valve abnormalities; - Dysphagia [18].
MPS IV	*GALNS*, *GLB1*	Morquio A,B	KS, CS	IVA - Short stature; - Kyphosis; - Pectus carinatum; - Joint abnormalities; - Mortality between 20 and 30 years old [19]. IVB Symptoms similar to IVA.
MPS VI	*ARSB*	Marateaux-Lamy	DS	- Impaired vision; - Severe orthopedic problems; - Growth impairment; - Hepatosplenomegaly; - Reduced pulmonary function; - Heart valve changes; - Carpal tunnel syndrome; - Spinal cord compression [20].
MPS VII	*GUSB*	Sly	DS, HS, CS	- Hydrops fetalis; - Craniofacial dysmorphia; - Hepatosplenomegaly; - Short stature; - Inguinal and umbilical hernias [16].
MPS IX	*HYAL1*	Natowicz	HA	- Short stature; - Periarticular masses; - Erosion of hip joint [21].
MPS X	*ARSK*		DS	- Short stature; - Skeletal abnormalities; - Cardiac disorders; - Ophthalmological abnormalities [22].
MPSPS	*VPS33A*		HS, DS	- Similar to other MPS types but include hematopoietic abnormalities and renal failure; - Heart defects; - Barrel-shaped chest; - Hepatosplenomegaly; - Skeletal dysplasia; - Psychomotor retardation [23].

MPS: mucopolysaccharidoses; DS: dermatan sulfate; HS: heparan sulfate; HA: hyaluronic acid; KS: keratan sulfate; CS: chondroitin sulfate; H–S: Hurler–Sheie.

**Table 2:** Comparison of ENT symptoms in MPS in original articles.

Article	Total Number of Patients	ENT Manifestation	Number of Patients with ENT Manifestation (n/N; (%))
Gökdoğan et al., 2016 [24]	9 MPS I (3) MPS III (1) MPS IV (2) MPS VI (3)	Hearing loss	9/9 (100%)
Yeung et al., 2009 [11]	27	Recurrent respiratory infections	11/27 (41%)
		Otitis media	19/27 (70%)
		Upper airway obstruction	MPS I (79%) MPS II (100%) MPS III (50%) MPS IV (0%) MPS VI (50%) MPS VII (0%)
Gönüldaş et al., 2014 [12]	76	Otitis media with effusion	MPS I 8/8 (100% )MPS II 8/9 (88.9%) MPS III 17/23 (73.9%) MPS IV 11/13 (84.6%) MPS VI 19/21 (90.5%)
		Obstructive sleep apnea	MPS I 4/4 (100%) MPS II 5/5 (100%) MPS III 7/8 (87.5%) MPS IV 6/7 (85.7%) MPS VI 18/18 (100%)
Mesollela et al., 2013 [13]	20	Otitis media	MPS I 6/7 (85.7%) MPS II 6/6 (100%) MPS III 3/4 (75%) MPS IV 1/1 (100%) MPS VI 1/1 (100%)
		Hearing loss	MPS I 7/7 (100%) MPS II 4/6 (66.7%) MPS III 2/4 (50%) MPS IV 1/1 (100%) MPS VI 1/1 (100%)
		Adenotonsillar hypertrophy	MPS I 3/7 (42.9%) MPS II 3/6 (50%) MPS III 2/4 (50%) MPS IV 0/1 (0%) MPS VI 1/1 (100%)
		Laryngitis/Pharyngitis/Rhinitis	MPS I 7/7 (100%) MPS II 6/6 (100%) MPS III 4/4 (100%) MPS IV 1/1 (100%) MPS VI 1/1 (100%)
		Obstructive sleep apnea	MPS I 2/7 (29%) MPS II 3/6 (50%) MPS III 3/4 (75%) MPS IV 0/1 (0%) MPS VI 0/1 (0%)
Lee et al., 2023 [25]	MPS IVA 15	Adenoid hypertrophy	12/15 (80%)
		Macroglossia	14/15 (93.3%)
		Tonsillar hypertrophy	12/15 (80%)
Cohen et al., 2017 [18]	MPS III 34	Hearing loss	20/34 (58.8%)
		Adenotonsillar hypertrophy	15/34 (44.1%)
		Recurrent respiratory tract infections	15/34 (44.1%)
		Obstructive sleep apnea (OSA)	13/34 (38.2%)
Torres et al., 2019 [26]	23	Otalgia	10/23 (43.5%)
		Airway disorders	13/23 (56.5%)
		Snoring, respiratory distress at night and/or sleep apnea	15/23 (65.2%)
		Speech delay	12/23 (52.2%)
		Suspected hearing loss	9/23 (39.1%)
		At least one of the above	20/23 (87%)
Lee et al., 2021 [27]	42	Hearing loss	MPS I 5/7 MPS II 15/15 (100%) MPS III 4/8 (50%) MPS IV 8/9 (88.9%) MPS VI 3/3 (100%)
		Adenotonsillar hypertrophy	MPS I 3/7 MPS II 9/15 MPS III 4/8 (50%) MPS IV 7/9 MPS VI 2/3 (66.7%)
		Laryngitis/pharyngitis/rhinitis	MPS I 7/7 (100%) MPS II 15/15 (100%) MPS III 8/8 (100%) MPS IV 9/9 (100%) MPS VI 3/3 (100%)
		Obstructive sleep apnea	MPS I 4/7 MPS II 3/15 (20%) MPS III 1/8 (12.5%) MPS IV 3/9 (33.3%) MPS VI 3/3 (100%)
Mendelsohn et al., 2010 [28]	MPS II 527	Tympanostomy	271/527 (51.4%)
		Adenoidectomy	261/527 (49.5%)
		Tonsillectomy	187/527 (35.5%)
		Dental procedure	74/527 (14%)
		Tracheotomy	23/527 (4.4%)
de Mello et al., 2020 [29]	30 MPS I 6 MPS II 8 MPS III 2 MPS IV 3 MPS VI 11	Snoring	24/30 (80%)
		Sleep Apnea	16/30 (53.34%)
		Stridor	5/30 (16.67%)
		Dyspnea	8/30 26.67%
Morimoto et al., 2014 [30]	35 MPS I 5 MPS II 25 MPS III 2 MPS IV 2 MPS VI 1	Deformity of the laryngeal architecture	23/35 (65.71%)
		Abnormal morphology at the Th1 level in tracheal lumen	17/35 (48.57%)
Pal et al., 2015 [31]	MPS I 61	Sleep disordered breathing	68%
John et al., 2011 [32]	MPS VI 28	Obstructive sleep apnea	24/28 (85.1%)
Leighton et al., 2001 [33]	26 MPS I 10 MPS II 6 MPS III 4 MPS IV 4 MPS VI 2	Upper airway obstruction	23/26 (88.5%)
		Obstructive sleep apnea	17/26 (65.4%)
Santamaria et al., 2007 [34]	11 MPS I 2 MPS II 3 MPS III 1 MPS IV 4 MPS VI 1	Obstructive sleep apnea	6/11 (54%)
		Adenoid hypertrophy	11/11 (100%)
Pereira et al., 2016 [35]	MPS VI 11	Airways obstruction	11/11 (100%)
		Obstructive sleep apnea	9/11 (81.8%)
		hypertrophy of the nasal turbinates	9/11 (81.8%)
		severe infiltration in the supraglottic region	7/11 64%
Facchina et al., 2018 [36]	MPS IV 16	Obstructive sleep apnea	11/16 (69%)
Ahn et al., 2019 [37]	124 MPS I 14 MPS II 91 MPS III 2 MPS IV 14 MPS VI 3	Hearing loss	84/124 (67.6%)
		Middle ear effusion	83/124 (67.2%)
		Tympanic membrane perforation	15/124 (12.1%)
		Ventilation tube insertion	15/124 (12.1%)
		Retracted tympanic membrane	11/124 (8.6%)
Lin et al., 2014 [38]	39 MPS I 3 MPS II 21 MPS IV 9 MPS VI 6	Hearing loss	33/39 (85%)
Keilmann et al., 2012 [39]	MPS II 554	Otitis (either acute otitis media or chronic otitis media)	401/554 (72.4%)
		Hearing loss	373/554 (67.3%)
		Insertion of ventilation tubes	272/554 (49.6%)
		Adenoidectomy	267/554 (47.4%)
		Hearing aids	225/554 (40.6%)
		Otorrhea	185/554 (33.4%)
		Tympanic membrane perforation	66/554 (11.9%)
		Vertigo	15/554 (2.7%)
		Tinnitus	12/554 (2.2%)
da Silveira et al., 2018 [40]	53 MPS I 10 MPS II 3 MPS III 1 MPS IV 4 MPS VI 12	Hearing loss	51/53 (96.2%)
Nagao et al., 2019 [41]	MPS IV 14	Hearing loss	12/14 (85.71%)
		Multiple ear infection	13/14 (92.86%)
		Chronic ear infection	5/14 (35.71%)
Bicalho et al., 2021 [42]	24 MPS I 2 MPS II 6 MPS III 1 MPS IV 1 MPS VI 14	Hearing loss	
			1/2 (50%)
			4/6 (66.66%)
			0
			0
			4/14 (28.6%)
Cho et al., 2008 [43]	MPS II 19	Otitis media effusion	14/19 (73.7%)
		tympanic membrane perforation	1/19 (5%)
		Ventilation tubes	11/19 (57.7%)
		Adenoidectomy and/or tonsillectomy	9/19 (47.4%)
		Hearing loss	19/19 (100%)
Motamed et al., 2000 [44]	MPS I 2	Hearing loss	2/2 (100%)
	MPS II 2	Otitis media with effusion	2/2 (100%)
	MPS III 5	T-tube insertion	5/5 (100%)
Arn et al., 2015 [15]	MPS I 1041	Macroglossia	203/1041 (19.5%)
		Enlarged tonsils	205/1041 (19.7%)
		Sleep disturbances	278/1041 (26.7%)
Thümler et al., 2012 [20]	MPS VI 9	ENT interventions	5/9 (55.6%)
Lenka et al., 2020 [2]	61 MPS I 15 MPS II 10 MPS III 17 MPS IV 15 MPS VI 4	Otitis media with effusion	MPS I (39%) MPS II (50%) MPS III (14%) MPS IV (46%) MPS VI (50%)
		Hearing loss	MPS I (50%) MPS II (70%) MPS III (33%) MPS IV (67%) MPS VI (50%)
		Upper airways obstruction	MPS I (92%) MPS II (90%) MPS III (65%) MPS IV (27%) MPS VI (0%)
		Acute otitis media	MPS I (39%) MPS II (90%) MPS III (35%) MPS IV (47%) MPS VI (50%)
		Chronic/recurrent rhinosinusitis	MPS I (85%) MPS II (70%) MPS III (93%) MPS IV (50%) MPS VI (25%)

## Data Availability

No new data were created or analyzed in this study.

## Supplementary Materials

The following supporting information can be downloaded at: https://www.mdpi.com/article/10.3390/brainsci14111085/s1.
