# Supplementary material for: Otorhinolaryngological Problems in Mucopolysaccharidoses: A Review of Common Symptoms in a Rare Disease
Source: Brain Sci. 2024 Oct 29;14(11):1085. doi: 10.3390/brainsci14111085 (PMC11591799; doi:10.3390/brainsci14111085)
Supplement: Supplementary file 1 [file brainsci-14-01085-s001.zip › brainsci-3248595-supplementary.pdf]

	Year	Country	Type
Lenka et al. 2020 [2]	2020	Czech Republic	Article
Simmons et al. 2005 [9]	2005	UK	Review
Yeung et al. 2009 [11]	2009	USA	Article
Gönüldaş et al. 2014 [12]	2014	Turkey	Article
Mesollela et al. 2013 [13]	2013	Italy	Article
Arn et al. 2015 [15]	2015	USA, UK	Article
Cohen et al. 2017 [18]	2017	UK	Article
Thümler et al. 2012 [20]	2012	Germany	Article
Lee et al. 2023 [25]	2023	Taiwan	Article
Gökdoğan et al. 2016 [24]	2016	Turkey	Article
Torres et al. 2019 [26]	2019	Brazil	Article
Lee et al. 2021 [27]	2021	Taiwan	Article
Mendelsohn et al. 2010 [28]	2010	USA	Article
de Mello et al. 2020 [29]	2020	Brazil	Article
Morimoto et al. 2014 [30]	2014	Japan	Article
Pal et al. 2015 [31]	2015	UK	Article
John et al. 2011 [32]	2011	Brazil	Article
Leighton et al. 2001 [33]	2001	UK	Article
Santamaria et al. 2007 [34]	2007	Italy	Article
Pereira et al. 2016 [35]	2016	Brazil	Article
Facchina et al. 2018 [36]	2018	Italy, France	Article
Ahn et al. 2019 [37]	2019	Korea	Article
Lin et al. 2014 [38]	2014	Taiwan	Article
Keilmann et al. 2012 [39]	2012	Germany	Article
da Silveira et al. 2018 [40]	2018	Brazil	Article
Nagao et al. 2019 [41]	2019	USA	Case report
Bicalho et al. 2021[42]	2021	**Brazil**	Article
Cho et al. 2008 [43]	2008	South Korea	Article
Motamed et al. 2000 [44]	2000	UK	Article
de Bode et al. 2022 [45]	2022	The Netherlands	A systematic review
Kubaski et al. 2020 [46]	2020	USA, Brazil	Review
Shinhar et al. 2004 [47]	2004	USA	Case series
Carneiro et al. 2021 [48]	2021	Brazil	Original aricle
De Santana Sarmento et al. 2015 [49]	2015	Mexico	Case report
Hampe et al. 2021 [50]	2021	USA	Review
Galimberti et al. 2018 [51]	2018	Italy	Review
Shapiro et al. 1985 [52]	1985		Case reports
Keilmann et al. 2015 [53]	2015	Germany	Article
Bianchi et al. 2018 [54]	2018	Italy	Review
Lin et al. 2013 [55]	2013	Taiwan	Article
Nayak et al. 1998 [56]	1998	India	Case reports
Tweedie et al. 2012 [57]	2012	UK	Article
Elwell et al. 2023 [58]	2023	USA	Article

Pal et al. 2018 [59]	2018	UK, Italy	Article
Soni-Jaiswal et al. 2014 [60]	2014	UK	Case report
Myer et al. 1991 [61]	1991	USA	Case report
Peters et al. 1985 [62]	1985		Article
Muhlebach et al. 2013 [63]	2013	USA	Article
Theroux et al. 2023 [64]	2023	USA	Article
Frauenfelder et al. 2023 [65]	2023	UK, Australia	Case report
Adachi et al. 1990 [66]	1990	USA	Case report
Kampmann et al. 2017 [67]	2017	Germany	Case report
Mierzwiński et al. 2006 [68]	2006	Poland	Case report
Akyol et al. 2019 [69]	2019	Turkey, USA, Japan, Brazil, Canada, Italy	Recommendations and guidelines
Jeong et al. 2006 [70]	2006	Republic of Korea	Article
Mitchell et al. 2016 [71]	2016	UK, USA, Colombia, Canada, Italy	Review
Karl et al. 2016 [72]	2016	Germany	Case report
Morehead et al. 1993 [73]	1993	USA	Case report
Pal et al. 2015 [74]	2015	UK	Systematic review
Berger et al. 2013 [75]	2013	USA	Review
Terai et al. 2020 [76]	2020	Japan	Review
Wolfberg et al. 2020 [77]	2020	USA, Japan	Review
Kariya et al. 2012 [78]	2012	USA	Article
Nagao et al. 2018 [79]	2018	USA	Article
Oghan et al. 2007 [80]	2007	Turkey	Case report
Souillet et al. 2003 [81]	2003	France	Article
van den Broek et al. 2020 [82]	2020	The Netherlands	Article
Muenzer et al. 2006 [83]	2006	USA	Article
Hong et al. 2012 [84]	2012	South Korea	Article
Parini et al. 2020 [85]	2020	Italy	Article
Tokic et al. 2006 [86]	2006	Republic of Croatia	Case report
